# Changing epidemiology of meticillin-resistant *Staphylococcus aureus* in 42 hospitals in the Dutch–German border region, 2012 to 2016: results of the search-and-follow-policy

**DOI:** 10.2807/1560-7917.ES.2019.24.15.1800244

**Published:** 2019-04-11

**Authors:** Annette Jurke, Inka Daniels-Haardt, Welmoed Silvis, Matthijs S. Berends, Corinna Glasner, Karsten Becker, Robin Köck, Alex W. Friedrich

**Affiliations:** 1 North Rhine-Westphalian Centre for Health, Section Infectious Disease Epidemiology, Bochum, Germany; 2 North Rhine-Westphalian Centre for Health, Department Health Promotion, Health Protection, Bochum, Germany; 3 Laboratory for Medical Microbiology and Public Health (LabMicTA), Hengelo, Netherlands; 4 Certe Medical Diagnostics and Advice, Groningen, Netherlands; 5 University of Groningen, University Medical Center Groningen, Department of Medical Microbiology and Infection Prevention, Groningen, Netherlands; 6 University Hospital Münster, University of Münster, Institute of Medical Microbiology, Münster, Germany; 7 University Hospital Münster, University of Münster, Institute for Hygiene, Münster, Germany; 8 Institute of Hygiene, DRK Kliniken Berlin, Berlin, Germany

**Keywords:** HAI surveillance, regional network for infection prevention, infection control, MRSA, Europe, healthcare-associated infections, bacterial infections, meticillin-resistant *Staphylococcus aureus*, antimicrobial resistance, hand hygiene, infection control, public health policy, surveillance, epidemiology

## Abstract

**Introduction:**

Meticillin-resistant *Staphylococcus aureus* (MRSA) is a major cause of healthcare-associated infections.

**Aim:**

We describe MRSA colonisation/infection and bacteraemia rate trends in Dutch–German border region hospitals (NL–DE-BRH) in 2012–16.

**Methods:**

All 42 NL–DE BRH (8 NL-BRH, 34 DE-BRH) within the cross-border network EurSafety Health-net provided surveillance data (on average ca 620,000 annual hospital admissions, of these 68.0% in Germany). Guidelines defining risk for MRSA colonisation/infection were reviewed. MRSA-related parameters and healthcare utilisation indicators were derived. Medians over the study period were compared between NL- and DE-BRH.

**Results:**

Measures for MRSA cases were similar in both countries, however defining patients at risk for MRSA differed. The rate of nasopharyngeal MRSA screening swabs was 14 times higher in DE-BRH than in NL-BRH (42.3 vs 3.0/100 inpatients; p < 0.0001). The MRSA incidence was over seven times higher in DE-BRH than in NL-BRH (1.04 vs 0.14/100 inpatients; p < 0.0001). The nosocomial MRSA incidence-density was higher in DE-BRH than in NL-BRH (0.09 vs 0.03/1,000 patient days; p = 0.0002) and decreased significantly in DE-BRH (p = 0.0184) during the study. The rate of MRSA isolates from blood per 100,000 patient days was almost six times higher in DE-BRH than in NL-BRH (1.55 vs 0.26; p = 0.0041). The patients had longer hospital stays in DE-BRH than in NL-BRH (6.8 vs 4.9; p < 0.0001). DE-BRH catchment area inhabitants appeared to be more frequently hospitalised than their Dutch counterparts.

**Conclusions:**

Ongoing IPC efforts allowed MRSA reduction in DE-BRH. Besides IPC, other local factors, including healthcare systems, could influence MRSA epidemiology.

## Introduction

Cross-border patient mobility is a priority in the European Union (EU), because the most accessible or appropriate care for citizens living in border regions may be available abroad. When, in 2013, the directive 2011/24/EU came into force, patients’ right to access healthcare in other Member States including reimbursement and medical follow-up in their respective home countries was entitled in an EU law for the first time. With this, cross-border cooperation in infection prevention and control (IPC) using comprehensive strategies is important [1]. 

Antimicrobial resistant (AMR) pathogens are a serious threat to public health in Europe, leading to increased healthcare costs, treatment failure and deaths. For invasive bacterial infections, prompt treatment with effective antimicrobial agents is essential and is one of the most effective interventions to reduce the risk of fatal outcomes [2]. Currently, the epidemiological situation is cause for concern especially with regard to AMR Gram-negative pathogens, e.g. characterised by carbapenem resistance (CR) [3]. However, the Gram-positive meticillin-resistant *Staphylococcus aureus* (MRSA) is still one of the most important causes of healthcare-associated infections due to AMR pathogens [3].

In 2017 in a consensus report of the European Centre for Disease Prevention and Control (ECDC), the European Food Safety Authority (EFSA) and the European Medicines Agency (EMA), the proportion of MRSA in invasive *S. aureus* infections was proposed as an indicator for surveillance of AMR pathogens in humans [4]. Although in 2016 the proportion of MRSA in invasive *S. aureus* infections in Europe reached its lowest level (13.7%) since the ECDC first presented population-weighted data for the EU in 2009, large inter-country variations (1.2 to 50.5%) remain in Europe [3]. For example, in the most populated German federal state, North Rhine-Westphalia (NRW), the incidence of MRSA bacteraemia per inhabitants was 32-fold higher compared with the Dutch neighbouring region with similar population size in 2009–10 [5].

The occurrence of MRSA still necessitates continuous surveillance and preparedness to optimise IPC to further decrease MRSA rates [6-9]. Since 1999, MRSA screening of various sites including at least nares, pharynx and wounds (if present) and additionally perineum or groin (in case of known previous carriage) before or at admission to hospitals is recommended in Germany, if patients have defined risk factors [10]. For MRSA carriers IPC measures including isolation in single rooms, barrier precautions and decolonisation therapies are also recommended [10,11]. Within the EU-funded community initiative INTERREG IIIA in 2006, all hospitals in the German Münsterland region, located directly at the Dutch–German border, started to establish a network to control MRSA – the EUREGIO MRSA net. They agreed to monitor the implementation of the IPC measures, harmonise local standards, exchange hospital utilisation data and MRSA data, perform molecular typing of MRSA isolates and establish regional benchmarks [12]. This ‘search-and-follow’ strategy was inspired from the ‘search-and-destroy’ policy implemented in Dutch hospitals since the 1980s. It aimed to improve application of the German national MRSA recommendations, the regional cooperation between hospitals, other healthcare facilities and public health authorities, as well as to create a more robust MRSA surveillance system [9,12-14]. Further to this strategy, screening for MRSA carriage among risk patients at hospital admission increased between 2009 and 2011 in these regional German hospitals and the nosocomial MRSA incidence density significantly decreased [15]. 

The cross-border IPC network cooperation, i.e. the Dutch−German web-based communication portal for handling MRSA problems for healthcare workers, patients and the public was continued from 2009 to 2015 within the INTERREG IVA funded project EurSafety Health-net. This enabled hospitals and nursing homes to acquire Euregional Quality and Transparency certificates. Moreover, since 2016, the collaboration was further prolonged within the INTERREG VA funded project EurHealth-1Health inter alia. Within this, the Dutch signaling meeting of the Hospital-acquired Infection and Antimicrobial Resistance Monitoring Group (SO-ZI/AMR) occurs in the German study region. 

Here, we analysed 2012 to 2016 MRSA surveillance data from Dutch and German border region hospitals (NL-BRH and DE-BRH) in the network in order to describe temporal and spatial trends of MRSA rates and find differences between these groups of hospitals. We also used the data to calculate the MRSA rates per inpatient and per patient days in both groups of hospitals and the MRSA rates per inhabitants in the patient catchment areas of NL-BRH and DE-BRH respectively in order to compare the two groups in relation to these parameters.

## Methods

### Setting

Within the EurSafety Health-net project (http://www.eursafety.eu/) the German part of the project region geographically comprised six districts in the Münsterland region (codes DEA33–35, DEA37, DEA38 and DE94B, level 3, according to the Nomenclature of Territorial Units for Statistics, NUTS [16]) and was inhabited by ca 1.73 million people [17]. The Dutch part comprised eight districts in the provinces of Groningen, Drenthe and in the region Twente-Achterhoek (codes NL111–113, NL131–133, NL213 and NL225) inhabited by ca 2.10 million people (Figure) [17]. Initially, there were 42 hospitals located in the Dutch–German region (reduced in 2015 to 41 due to a structural merging of two DE-BRH) treating ca 620,000 admitted patients (68.0% in the German part of the study region) with ca 3,900,000 patient days per year. All 34 (since 2015, 33) regional DE-BRH (9.5% of hospitals in NRW in 2016) and all eight regional NL-BRH (8.8% of hospitals in the Netherlands in 2016) took part in the project. Among the DE-BRH, 29 were acute care hospitals, one was a university hospital, one was a rehabilitation clinic and three hospitals were specialised in psychiatry, while the NL-BRH comprised one university- and seven acute care hospitals.

**Figure:**
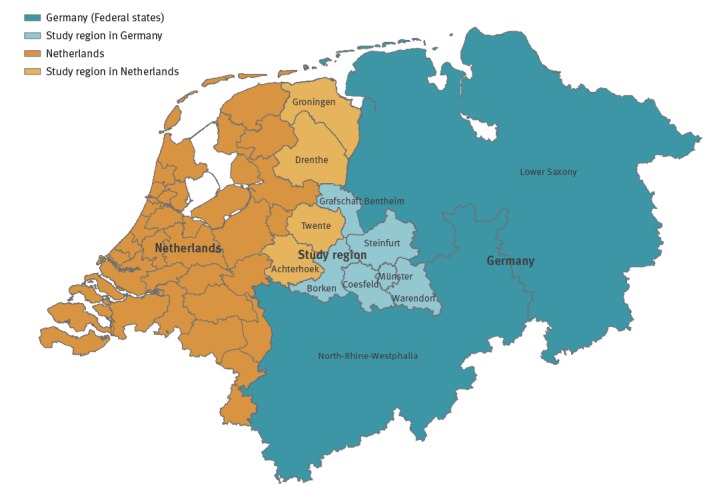
Location of the study region in the Netherlands and Germany, 2012–2016

The light orange area represents the study region within the Netherlands, including the Dutch regions East Groningen (NL111), Delfzijl and surroundings (NL112), rest of Groningen (NL113), North Drenthe (NL 131), South East Drenthe (NL132), South West Drenthe (NL133), Twente (NL213), Achterhoek (NL225). The light blue area represents the study region within Germany, including the Grafschaft Bentheim region (DE94B) and the Münsterland-region with the urban district Münster (DEA33) and the rural districts Borken (DEA34), Coesfeld (DEA35), Steinfurt (DEA37) and Warendorf (DEA38).

### Guidelines for patients at risk for meticillin-resistant *Staphylococcus aureus* and infection prevention and control measures

Both NL-BRH and DE-BRH implemented MRSA-related IPC measures according to their national guidelines and recommendations, issued by the Dutch Working Group on Infection Prevention (WIP) and the German Commission for Hospital Hygiene and Infection Prevention (KRINKO) at the Robert Koch-Institute, respectively [10,18]. Of note, the definitions of whom to screen at admission differed for NL-BRH and DE-BRH based on the national guidelines and recommendations (Table 1), as well as screening sites (DE-BRH: at least nose, pharynx, throat and wounds, if present, additionally perineum and groin swab, when indicated; NL-BRH: nasal-, throat- and perineum or rectum swab plus additional cultures depending on clinical signs) [10,19]. In all hospitals positive screenings or any other detection of MRSA was followed by single room isolation, contact precautions and decolonisation, if applicable. Pre-emptive isolation of patients with MRSA risk factors was performed according to local guidelines (in DE-BRH only for patients with previous MRSA carriage, for NL-BRH see Table 1. In both countries adherence to the MRSA-IPC guidelines- and recommendations was periodically checked by the responsible local public health authorities (Germany) and national health inspectorate (Netherlands). The implementation of other IPC measures in the participating hospitals, such as standards for the prevention of catheter-related bloodstream infections, was not planned or assessed within the project.

**Table 1:** Risk factors for MRSA carriage at admission according to Dutch and German MRSA guidelines, 2012–2016

MRSA carriage risk factors	Defined risk factors for MRSA screening according to national guideline/recommendation	
	Germany	Netherlands
(Previous) MRSA carriage or infection	Known MRSA carrier or previous MRSA carriage or infection	Known MRSA carrier (RMRSA)
		(Previous) MRSA carrier who underwent decolonisation, without three consecutive negative MRSA screening tests, taken at least 7 days apart (RMRSA)
		(Previous) MRSA carrier who underwent decolonisation, with three consecutive negative MRSA screening tests, taken at least 7 days apart, and is within 1 year follow-up after first negative MRSA test (RL)
Contact to MRSA positive person	Contact with another person with MRSA carriage or infection (same room)	Unprotected contact within the last 2 months: - Inside hospital: part of ring investigation (RH) - Outside hospital: household member, partner or caregiver of MRSA positive person (RH)^ a^ - Contact to MRSA positive healthcare worker, regardless of duration (RL)
		Persistent unprotected exposure: negative MRSA screening test within the last 3 months (RL)
Recent stay in other healthcare institution	Hospitalisation for > 3 days within the previous 12 months	Stayed in a foreign healthcare institution within the last 2 months (RH), and: - stayed more than 24 hours, or - stayed less than 24 hours plus at least one secondary risk factor (invasive procedure, chronic infections, persistent skin lesions, abscesses or furuncles) for MRSA carriage
		Stayed in a foreign healthcare institution more than 2 months ago plus at least one secondary risk factor (see above) for MRSA carriage (RL)
	Direct transfer of the patient from facilities in regions with known high MRSA prevalence (i.e. including all German healthcare facilities)	Previous hospitalisation within the last two months in a Dutch healthcare institution in a department with an ongoing MRSA outbreak (RH)
Haemodialysis patients	All haemodialysis patients^b^	- Patient usually dialysed abroad (i.e. ‘home dialysis center’ abroad), now dialysed in the Netherlands (i.e. guest dialysis patients) (RH) - Patient usually dialysed in the Netherlands (i.e. Dutch ‘home dialysis center’) dialysed abroad within the last two months (RL)
Contact to livestock	Regular professional direct contact to livestock (swine, cattle, poultry)^c^	-Contact to live pigs/meat calves/broilers^c^ regardless of whether this contact was professional or not and/or lives on a farm where these animals are kept (RH) - Persistent exposure: negative MRSA screening test within the last 3 months (RL)
Other risk factors	Chronic skin lesions	- Children adopted from abroad and living in the Netherlands (RH) - Stayed in a home for asylum seekers within the last two months (RH)^d^
	Need for long-term care plus one of the following risk factors^b^: (i) receipt of antibiotics during the previous 6 months, or (ii) presence of indwelling devices	

MRSA: meticillin-resistant *Staphylococcus aureus*; RMRSA/RH/RL: risk categories corresponding to different levels of isolation for inpatients.

^a^ Added in December 2012.

^b^ Modified in 2012–13: patient with two or more of the following risk factors: need for long-term care, receipt of any antibiotics during the previous six months, presence of indwelling devices, need for haemodialysis, skin lesions and burns.

^c^ Modified; in 2012–13 only swine.

^d^ Added in October 2015.

The levels of isolation for inpatients with risk categories were the following: (RMRSA) MRSA positive- or (RH) high-risk category patients in high-risk departments of the hospital (e.g. intensive care unit, haematology): single room isolation with contact- and airborne precautions. (RH) High-risk category patients who are not in high risk departments and who have an MRSA screening result available within 24 hours of admission: single room with contact precaution. (RL) Low-risk category: no isolation, awaiting new MRSA screening test results.

### Data collection

An MRSA case was defined as an inpatient who was colonised or infected with MRSA at admission or for nosocomial MRSA cases, after admission. A blood culture positive for MRSA, from a single inpatient and from a single hospital stay was qualified as MRSAB case. If an MRSA case, or MRSAB case, had several stays in a year, each hospital stay was counted as an MRSA case, or MRSAB case, in the surveillance. 

On both sides of the border, the collected surveillance data of inpatients (i.e. excluding outpatients) included the number of nasopharyngeal swabs performed for MRSA screening before or at admission, the numbers of MRSA cases (one isolate per patient per hospital stay) − in DE-BRH and in several NL-BRH MRSA cases were additionally classified as imported or nosocomial (i.e. nosocomial, if the case was detected ≥ 3 days after hospital admission unless the patient was a known MRSA carrier), the number of cases and the number of patient days. Additionally, in DE-BRH and in several NL-BRH the patient days of MRSA cases (i.e. the number of days, which an MRSA-positive patient spent in hospital) were also recorded. Moreover, the number of inpatients with a blood culture positive for MRSA (MRSAB, one isolate per patient case) and the number of *S. aureus* in blood cultures (one isolate per patient case) were assessed. The MRSA-surveillance data as described above were collected in all DE-BRH using a protocol adapted from the national German Nosocomial Infections Surveillance System (MRSA-KISS [20]); see Supplement Table S1). For cross-border analysis, the laboratories serving for all NL-BRH provided retrospectively collected data for the period 2012 to 2016, according to the same protocol. 

### Ethical statement

Ethical approval was asked from ethical committee at the University Medical Center Groningen (UMCG) and approval was not necessary for this study.

### Data analysis

We analysed the surveillance data of 5 years (2012–16) and calculated the following parameters: (i) screening rate (nasopharyngeal swabs for MRSA/100 inpatients), (ii) MRSA incidence (MRSA cases/100 inpatients), (iii) percentage of MRSA isolates per all *S. aureus* isolates detected in blood cultures, (iv) incidence density of MRSA isolates detected from blood cultures (MRSAB cases/100,000 patient days), (v) nosocomial MRSA incidence density (nosocomial MRSA-cases/1,000 patient days), (vi) length of stay in hospital (number of patient days/inpatients, (vii) length of stay in the hospital of MRSA cases (number of patient days of MRSA cases/MRSA cases). We calculated the mean annual numbers of inpatients per 100 inhabitants and of patient days per 100 inhabitants of the patient catchment area of NL-BRH and DE-BRH. Furthermore, we calculated the mean annual number of nasopharyngeal swabs performed for MRSA screening before or at admission to hospital per 100 inhabitants in the patient catchment area of the regional hospitals (DE-BR and NL-BR) as well as of inpatient MRSA cases per 1,000 inhabitants and the MRSAB/1,000,000 inhabitants using our surveillance data of inpatients (i.e. excluding outpatients). The number of inhabitants were assessed from the official statistical database [17].

Time trends of MRSA parameters were analysed by Friedman tests. The percentage of nosocomial MRSA cases on all MRSA cases was assessed by Cochran–Armitage test of linear trend. The cross-border regional comparisons were analysed using Wilcoxon rank sum test. All statistical analyses were done using SAS 9.4 software (SAS Institute Inc., Cary, United States); p < 0.05 was considered significant. Results of significance tests were discarded if the software displayed an alert due to more than 10% of missing values in the respective dataset. The map was made using RegioGraph10 (GFK Geomarketing GmbH, Bruchsal, Germany).

## Results

### Trend and cross-border comparison of meticillin-resistant *Staphylococcus aureus* rates

The total numbers of MRSA cases (detected in DE-BRH and NL-BRH are shown in Table 2. In both DE-BRH and NL-BRH the median nasopharyngeal MRSA screening rate increased significantly between 2012 and 2016 (Table 3). Overall, the median screening rate was 14 times higher in DE-BRH than in NL-BRH (p < 0.0001, Table 4).

**Table 2:** Numbers of meticillin-resistant *Staphylococcus aureus* cases documented in all study hospitals in the German region of Münsterland and the Dutch regions of Twente-Achterhoek, Drenthe and Groningen, 2012–2016 (n = 42 hospitals)^a^

Region, country (number of BRH)	MRSA cases	Year									
		2012		2013		2014		2015		2016	
		n	%	n	%	n	%	n	%	n	%
Münsterland, Germany (34 DE-BRH)^a^	MRSA (total)	4,453	100.0	4,481	100.0	4,391	100.0	4,418	100.0	4,122	100.0
	Nosocomial MRSA cases^b^	430	9.7	361	8.1	316	7.2	266	6.0	260	6.3
	MRSAB cases	72	NA	93	NA	53	NA	56	NA	60	NA
Twente-Achterhoek/Drenthe/Groningen, Netherlands (8 NL-BRH)^a^	MRSA (total)	216	100.0	295	100.0	308	100.0	321	100.0	327	100.0
	MRSA cases with known status imported or nosocomial^b^	77	35.6	133	45.0	134	43.5	143	44.5	133	40.7
	Nosocomial MRSA cases^b^	10	13.0^b^	16	12.0^b^	22	16.4^b^	18	12.6^b^	14	10.5^b^
	MRSAB cases	5	NA	12	NA	11	NA	12	NA	3	NA

DE-BRH: German border region hospitals; MRSA: meticillin-resistant *Staphylococcus aureus*; MRSAB: MRSA isolated from blood cultures; NA: not applicable; NL-BRH: Dutch border region hospitals.

^a^ From 2015 onwards, the number of DE-BRH was reduced to 33. This implies that the total number of hospitals in the study region became 41 after 2015.

^b^ Data about the classification of cases as ‘nosocomial’ or ‘imported’ were only available for German hospitals, Dutch hospitals in the Twente-Achterhoek region and since 2013, for one hospital in Groningen, Netherlands. The given percentages refer to the percentages of nosocomial cases among those MRSA cases for whom this information was documented.

**Table 3:** Annual medians of meticillin-resistant *Staphylococcus aureus* parameters in all study hospitals in the German region Münsterland and the Dutch regions of Twente-Achterhoek, Drenthe and Groningen, 2012–2016 (n = 42 hospitals)^a^

Region, country (number of BRH)	MRSA parameter	Year(s)					
		2012	2013	2014	2015	2016	2012–16
		Median (IQR)	Median (IQR)	Median (IQR)	Median (IQR)	Median (IQR)	p value
Münsterland, Germany (34 DE-BRH)^a^	Nasopharyngeal swabs for MRSA screening per inpatients (%)	37.7 (31.6–54.7)	40.3 (33.9–51.1)	43.6 (31.7–55.1)	44.1 (35.8–57.1)	47.4 (38.4–63.5)	0.0006
	MRSA cases/100 inpatients	1.1 (0.8–1.6)	1.0 (0.7–1.3)	1.0 (0.7–1.4)	1.1 (0.8–1.3)	0.9 (0.8–1.3)	0.0814
	MRSAB/SAB (%)	12.5 (2.9–25.0)	14.3 (6.3–25.0)	10.5 (4.0–25.0)	9.8 (2.6–28.6)	5.0 (0.0–10.7)	0.0959
	MRSAB/100,000 patient days	1.3 (0.0–2.8)	2.6 (0.0–4.9)	1.7 (0.0–2.7)	1.2 (0.0–3.0)	1.5 (0.0–2.8)	0.4272
	Nosocomial MRSA cases/1,000 patient days^b^	0.11 (0.06–0.18)	0.09 (0.04–0.16)	0.09 (0.03–0.14)	0.08 (0.03–0.12)	0.07 (0.02–0.13)	0.0184
Twente-Achterhoek/Drenthe/Groningen, Netherlands (8 NL-BRH)	Nasopharyngeal swabs for MRSA screening per inpatients (%)	2.05 (0.65–4.10)	3.65 (0.65–4.60)	2.80 (0.65–4.65)	3.55 (0.60–7.20)	5.45 (0.85–10.05)	0.0188
	MRSA cases/100 inpatients	0.11 (0.09–0.13)	0.13 (0.10–0.14)	0.12 (0.09–0.16)	0.13 (0.10–0.15)	0.17 (0.11–0.25)	0.0816
	MRSAB/SAB (%)	0.7 (0.0–3.4)	1.6 (0.0–4.3)	1.0 (0.0–5.0	1.9 (0.0–4.3)	0.0 (0.0–1.3)	0.1679
	MRSAB/100,000 patient days	0.3 (0.0–1.3)	0.6 (0.0–1.9)	0.6 (0.0–2.0)	1.0 (0.0–1.9)	0.0 (0.0–0.6)	0.0620
	Nosocomial MRSA cases/1,000 patient days^b^	0.03 (0.02–0.04)	0.025 (0.020–0.035)	0.035 (0.030–0.055)	0.030 (0.020–0.045)	0.015 (0.005–0.030)	0.3532^b^

BRH: border region hospitals; DE-BRH: German BRH; IQR: interquartile range; MRSA: meticillin-resistant *Staphylococcus aureus*; MRSAB: MRSA isolated from blood cultures; NL-BRH: Dutch BRH; SAB: *S. aureus* isolated from blood cultures.

^a ^Since 2015 the number of DE-BRH was reduced to 33. This implies that the total number of hospitals in the study region became 41 after 2015.

^b^ Only available for German hospitals, Dutch Twente/Achterhoek hospitals and, since 2013, for one Groningen hospital, Netherlands.

**Table 4:** Meticillin-resistant *Staphylococcus aureus* parameters in all study hospitals in the German region of Münsterland and the Dutch regions of Twente-Achterhoek, Drenthe and Groningen, 2012–2016 (n = 42 hospitals)^a^

Parameter	Münsterland, Germany (34 DE-BRH)^a^		Twente-Achterhoek, Drenthe, Groningen, Netherlands (8 NL-BRH)^a^		p value (median comparison)
	Mean^b^	Median (IQR)	Mean^b^	Median (IQR)	
Nasopharyngeal swabs for MRSA screening/100 inpatients (%)	50.2	42.3 (33.8–56.8)	3.9	3.0 (0.6–5.1)	< 0.0001
MRSA cases of colonisation and/or infection/100 inpatients	1.04	1.04 (0.77–1.36)	0.15	0.14 (0.10–0.20)	< 0.0001
MRSAB/SAB (%)	9.8	10.2 (3.0–21.5)	1.5	0.3 (0.0–4.0)	< 0.0001
MRSAB/100,000 patient days	2.30	1.55 (0.00–3.53)	0.83	0.26 (0.00–1.72)	0.0041
Nosocomial MRSA cases/1,000 patient days^c^	0.11	0.09 (0.03–0.14)	0.03	0.03 (0.02–0.04)	0.0002
LOS in the hospital	6.9	6.8 (5.7–9.4)	5.3	4.9 (4.7–5.4)	< 0.0001
LOS of MRSA patients^d^	11.4	11.1 (8.5–14.2)	12.1	11.7 (5.6–17.5)	0.8774

DE-BRH: German border region hospitals; IQR: interquartile range; LOS: length of stay; MRSA: meticillin-resistant *Staphylococcus aureus*; MRSAB: MRSA isolated from blood cultures; NL-BRH: Dutch border region hospitals; SAB: *S. aureus* isolated from blood cultures.

^a^ Since 2015 the number of DE-BRH was reduced to 33. This implies that the total number of hospitals in the study region became 41 after 2015.

^b^ Pooled mean value.

^c^ Only available for German hospitals, Dutch Twente-Achterhoek hospitals and, since 2013, for one Groningen hospital, Netherlands.

^d^ Only available for German hospitals, two Dutch Twente-Achterhoek and two Groningen hospitals.

The median MRSA incidence remained stable over time at both sides of the border (Table 3), but was more than seven times higher in DE-BRH than in NL-BRH (p < 0.0001) (Table 4). The median percentage of MRSA on *S. aureus* blood culture isolates decreased from 12.5% in 2012 to 5.0% in 2016 in DE-BRH (p = 0.0959), while it remained stable in NL-BRH (p = 0.1679) (Table 3), but was more than 34 times higher in DE-BRH (p = 0.0001) (Table 4). The median of MRSAB per 100,000 patient days remained stable over time in DE-BRH (p = 0.4272) and NL-BRH (p = 0.0620) (Table 3) and was six fold greater in DE-BRH than in NL BRH (p = 0.0041) (Table 4). The percentages of nosocomial cases on all MRSA cases (Table 2) decreased significantly in DE-BRH (p < 0.0001), but did not change in NL-BRH (p < 0.6474). Over the study period the median nosocomial MRSA incidence-density decreased significantly in DE-BRH (p = 0.0184) (Table 3), but did not change in NL-BRH (p = 0.3532) and was approximately three times higher in DE-BRH than in NL-BRH (p = 0.0002) (Table 4).

### Cross-border comparison of healthcare utilisation

We compared the available data on healthcare utilisation in DE-BRH and NL-BRH. The median length of stay (LOS) in the hospital was 6.8 days in DE-BRH compared with 4.9 days in NL-BRH (p < 0.0001) (Table 4); LOS of MRSA patients was similar in DE-BRH vs NL-BRH (11.1 days vs 11.7 days; p = 0.8774) (Table 4). The hospitalisation rate was 24.3 inpatients/100 inhabitants annually in the patient catchment area of DE-BRH, almost thrice the rate in the NL-BRH’s catchment area (9.27/100). To put this difference in healthcare utilisation into context, we calculated the mean annual number of nasopharyngeal MRSA screening swabs before or at admission to hospital per 100 inhabitants in the German border region (DE-BR) vs the Dutch border region (NL-BR) (12.2 vs 0.36). Additionally, we compared the MRSA surveillance data of inpatients (i.e. excluding outpatients) in the patient catchment area of DE-BRH and NL-BRH. The calculated the number of inpatient MRSA cases per 1,000 inhabitants in DE-BR and NL-BR were 2.52 vs 0.14. Furthermore, the calculated MRSAB/1,000,000 inhabitants in DE-BR and NL-BR was 38.4 vs 4.09 (Table 5).

**Table 5:** Calculated parameters in the patient catchment area of all study hospitals in the German region of Münsterland and Dutch regions of Twente-Achterhoek, Drenthe and Groningen, 2012–2016 (n = 42 hospitals)^a^

Parameter	Münsterland, Germany (DE-BR)^a^ Mean^c^	Twente-Achterhoek, Drenthe, Groningen, Netherlands (NL-BR)^b^ Mean^c^
Inpatients/100 inhabitants	24.3	9.27
Patient days/100 inhabitants	167.2	49.0
Nasopharyngeal swabs for MRSA screening before or at admission to hospital/100 inhabitants	12.2	0.36
Inpatient MRSA cases of colonisation and/or infection/1,000 inhabitants	2.52	0.14
MRSAB/1,000,000 inhabitants	38.4	4.09

DE-BR: German border region; MRSA: meticillin-resistant *Staphylococcus aureus*; MRSAB: MRSA isolated from blood cultures.

^a^ Patient catchment area of 34 (since 2015: 33) German border region hospitals. This implies that the total number of hospitals in the study region became 41 after 2015.

^b^ Patient catchment area of eight Dutch border region hospitals.

^c^ Pooled mean value.

## Discussion

As patients in the EU have the right to healthcare across the borders of Member States (EU directive 2011/24/EU), it is of interest to compare the quality of care, safety standards and risks of nosocomial infection by AMR pathogens between EU countries. In this respect, the cross-border systematic and continuous MRSA surveillance is one of the cornerstones to ensure equal quality of healthcare [21]. 

Our study revealed significant differences between Dutch and German hospitals (Table 4). The median MRSA-incidence in DE-BRH was more than seven times higher compared with NL-BRH. We also found that the median MRSA percentage of *S. aureus* detected in blood cultures was more than 34 times higher in DE-BRH than in NL-BRH (Table 4). The incidence density of MRSAB was six times higher in DE-BRH (Table 4) and there were nine times more MRSAB per 1,000,000 inhabitants for the patient catchment area of DE-BRH compared with NL-BRH (Table 5).

According to the ECDC, differences in the occurrence of AMR pathogens between European countries are most likely caused by differences in healthcare utilisation, antimicrobial use and IPC practices [3]. 

Concerning healthcare utilisation in our context, we found that inhabitants in the German part of the study region were almost three times as often hospitalised (Table 5) and had a significantly longer LOS than patients on the Dutch part (Table 4). This may be due to socioeconomic factors or a different organisation of ambulatory healthcare. 

While antimicrobial consumption was not the focus of the current study, NRW has been reported as the region in Germany with the highest antimicrobial consumption in outpatients (19.2 daily defined doses (DDD/1,000 inhabitants) [22]. In this respect, the MRSA incidence in DE-BRH was slightly above the incidences in German hospitals participating in the nationwide surveillance system MRSA-KISS [20]. The antimicrobial consumption level in NRW seems to be also considerably higher than in the Netherlands (10.39 DDD/1,000 inhabitants) [23], not only in terms of total antibiotics consumed, but also for the oral use of second-generation cephalosporins. Promoting rational regional antibiotic use is therefore one of the major goals in the INTERREG VA project EurHealth-1Health (http://www.eurhealth-1health.eu/). 

For MRSA IPC, the recommendations in Germany and the corresponding guidelines in the Netherlands were comparable regarding the measures performed for MRSA carriers [10,18]. However, there were differences between the two countries in identifying people at risk of MRSA infection/colonisation [10,18]. In this study, we found that the DE-BRH performed 14 times more nasopharyngeal screening swabs for MRSA than their Dutch counterparts. 

The higher screening rates on the German side of the border may be ascribed to the fact that in German IPC recommendations, previous hospitalisation in Germany is a risk factor for MRSA carriage. This constitutes a main difference in defined risk factors between Dutch- and German MRSA IPC guidelines, whereby Dutch guidelines mostly consider screening for patients previously hospitalised outside the Netherlands (Tables 1 and 3) [14,24]. In this respect, we observed that although the densities of nosocomial MRSA cases were lower in NL-BRH than in DE-BRH (Table 3), the proportion of nosocomial MRSA cases among all MRSA detected was slightly higher in the Dutch hospitals (Table 2). The reason for this remains unclear, but it might be speculated that a larger proportion of MRSA carriers in the Netherlands had no risk factors for MRSA and were hence not screened at admission.

Another explanation for screening rate differences between the two countries may be distinct underlying epidemiological situations regarding MRSA. For example, the MRSA prevalence is higher in the population in Germany than that in patients at hospital admission in the Netherlands (0.7% vs. 0.13%) [25,26]. Moreover in the German part of the study region, a possible additional MRSA burden due to the exceptionally frequent occurrence of livestock-associated MRSA might have an effect [27,28]. 

The screening and IPC measures in the DE-BRH appeared to be nevertheless appropriate. In 2006, in the project region excluding Groningen and Drenthe (Figure), investigations evaluating the numbers of patients with MRSA risk factors at admission to German hospitals demonstrated that ca 35.6% of patients had a risk factor requiring screening [29]. A corresponding level of screening was implemented by DE-BRH during the study period 2009–11 [15]. This level remained high in the 2012–16 period (Table 3), indicating a very good implementation of the screening standards. 

About 1% of all patients admitted in DE-BRH carried MRSA, which corresponds well to results of investigations evaluating the prevalence of MRSA carriage in the regional general, non-hospitalised population in 2012 [25]. In terms of difference with the Netherlands, this has for consequence that it is more expensive to provide isolation capacities for ca 1.0% of inpatients with MRSA in DE-BRH vs 0.15% in NL-BRH. Moreover, the higher MRSA incidence in DE-BRH could lead to a higher probability for nosocomial MRSA cases as they are not completely avoidable [30-32]. 

From 2012 to 2016 however, the nosocomial MRSA incidence density in DE-BRH decreased significantly, a trend already observed from 2009 to 2011 [15]. Moreover, the nosocomial MRSA incidence density (Table 3) appeared to be below the densities reported for hospitals participating in the nationwide surveillance system MRSA-KISS (median nosocomial MRSA cases per 1,000 patient days in DE-BRH/MRSA KISS, 2012–16: 0.11/0.14, 0.09/0.12, 0.09/0.10, 0.08/0.09, 0.07/0.08) [15,20]. This may indicate the successful implementation of concerted IPC standards in DE-BRH in the EurSafety Health-net network [15]. 

We also observed for that the difference of the incidence of MRSA bacteraemia per inhabitants between the German and Dutch border region (38.4 vs 4.09 per 1,000,000) was apparently smaller than calculated in a previous study, which used 2009 Dutch and 2010 German data respectively to derive the difference between NRW and the Netherlands (57.6 vs 1.8 per 1,000,000) [5]. In addition, according to the population-based German mandatory notification system for invasive MRSA infections (SurvStat) from 2012 to 2016, 40.7 MRSA isolates were detected in blood or cerebrospinal fluid per 1,000,000 inhabitants in the German project region [33], which is lower compared with data from the federal state of NRW (70.3 per 1,000,000 inhabitants) as well as from Germany (47.9 per 1,000,000 inhabitants) [34]. 

Comparing our results with those of other German laboratories participating in a voluntary, national surveillance system (ARS) [35], revealed that, for each year of the period 2012–16 the median percentage of MRSA in *S. aureus* from blood cultures was lower in DE-BRH than in other laboratories in western Germany (DE-BRH/ARS-region west (NRW), 2012–16: 12.5%/19.0%, 14.3%/15.0%, 10.5%/13.5%, 9.8%/13.3%, 5.0%/12.0%) (Table 3), as well as below the middle lower range of the EU/European Economic Association (EEA) population-weighted mean between 18.8% in 2012 and 13.7% in 2016 [3,34,36]. 

In contrast, the mean MRSA percentage of *S. aureus* detected in blood culture during 2012–16 was higher (1.5% vs 1.3%) in NL-BRH compared with Dutch national data of Infectious Disease Surveillance Information System for Antibiotic Resistance, (ISIS-AR) covering data of 52% of diagnostic laboratories [37].

As typical for all passive surveillance systems, bias due to differences in reporting behaviour cannot be excluded and is a limitation of this study. However, as MRSA surveillance in DE-BRH started in 2007, a stabilised compliance in reporting can be assumed for the period from 2012–16. The higher number of MRSA cases per inhabitants on the German side compared with the Netherlands is biased if there is more than one episode of MRSA detection per year for one individual patient among the number of cases. Also the inclusion of three psychiatric hospitals and one rehabilitation clinic, which have usually longer average lengths of stay, may have prolonged hospital stay in the DE-BRH. However, the data are in accordance with German-wide assessment systems. The clinical relevance of MRSA isolates detected in blood cultures is undisputable, but variations in blood culture diagnostics (e.g. frequency, performance) may result in bias when comparing MRSA percentages of *S. aureus* blood culture isolates between different countries [38]. A limitation of the study design is that the implementation of IPC standards, which are not directly targeted to control MRSA, such as bundles to prevent central-line-associated bloodstream infections (CLABSI), was not assessed and compared in the participating hospitals. Hence, changes of the incidence of MRSA bacteraemia could also be attributable to improvements in CLABSI prevention or other IPC standards.

This study on MRSA covering all hospitals across part of a European border as well as hospitals of all three care-categories demonstrated that routine MRSA surveillance may be helpful to monitor trends of MRSA parameters, to compare the MRSA rates and to indicate needs for further improvement to reach low MRSA rates EU-wide. Our results supplement the European and national surveillance systems. Ongoing efforts in MRSA prevention are recommended, including all healthcare sectors, especially with focus on One Health [39-42]. Moreover, cross-border surveillance should be extended to other multidrug-resistant organisms, such as CR *Enterobacteriaceae* in the future. 

## Supplementary Data

63000 Supplement
