# Supplementary material for: Changing epidemiology of meticillin-resistant Staphylococcus aureus in 42 hospitals in the Dutch–German border region, 2012 to 2016: results of the search-and-follow-policy
Source: Euro Surveill. 2019 Apr 11;24(15):1800244. doi: 10.2807/1560-7917.ES.2019.24.15.1800244 (PMC6470371; doi:10.2807/1560-7917.ES.2019.24.15.1800244)
Supplement: Supplement [file 1800244_JURKE_Supplement.pdf]

Protocol: Surveillance of Methicillin-resistant *Staphylococcus aureus* (MRSA) in inpatients of regional hospitals within the EurSafety Health-net- and EurHealth-1Health-project

‘This supplementary material is hosted by *Eurosurveillance* as supporting information alongside the article “Changing epidemiology of meticillin-resistant Staphylococcus aureus in 42 hospitals in the Dutch–German border region, 2012 to 2016: results of the search-and-follow-policy” on behalf of the authors who remain responsible for the accuracy and appropriateness of the content. The same standards for ethics, copyright, attributions and permissions as for the article apply. *Eurosurveillance* is not responsible for the maintenance of any links or email addresses provided therein.’
https://www.eurosurveillance.org/for-authors (supplementary material).

Background: Besides being a common cause of skin-, soft tissue- and bone-infections, Methicillin-resistant *Staphylococcus aureus* (MRSA) is one of the major causes of bloodstream infections in Europe (1).

AIM: Every hospital must implement measures to prevent MRSA locally. The AIM of this MRSA surveillance protocol for inpatients is to assess structured data about the occurrence of MRSA and the implementation of MRSA-related prevention strategies in acute care hospitals participating in a regional network in order to draw conclusions about the efficiency of the MRSA management within the network.

Methods: The surveillance protocol provides specifications and definitions in order to standardize data collection and enable regional analysis and benchmarking. The surveillance protocol shall be implemented in hospitals participating in a regional prevention network organized by public health authorities in the Dutch-German border area within the project Eurhealth-1Health funded within the framework of the INTERREG Va program of the European Union (http://www.eurhealth-1health.eu/). As the aim of this network is to harmonize local standards for MRSA prevention and control, the surveillance can be used to describe and analyze regional differences and trends in MRSA colonization and infection and compare the regional MRSA-rates to national or international surveillance data.

All MRSA cases of participating hospital are recorded in a MRSA public health report. Data must be recorded for the entire hospital. Only MRSA cases among inpatients are included in the report (see specifications below). The hospitals have to communicate descriptive parameters (structure and process parameters of the entire hospital, e.g. number of beds) and to participate in validation measures. The hospitals submit the MRSA public health report annually via the local health department to the EurHealth-1Health coordination center, irrespectively of whether they take part in other surveillance systems. The data of all participating hospitals are analyzed and an anonymized feedback report is provided by the coordination center. The obligatory specifications have to be used strictly. Data are usually recorded by every hospital, for example the number of patient-days, thus surveillance does not represent an increase in workload.

The surveillance data includes the number of nasopharyngeal swabs performed for MRSA screening, the number of MRSA cases (colonization and infection, one isolate per patient case; if identical patients are admitted multiple times within the surveillance period, every inpatient case is included), classified as community-onset “imported” (detected in a specimen obtained <3 days after admission) or hospital-onset “nosocomial” (detected in a specimen ≥3 days after admission), the total number of inpatient cases and the total number of patient days (for all inpatients in the hospital for the respective surveillance period), as well as the number of patient days of MRSA cases included in the

report (i.e. days, in which MRSA-positive patients were hospitalized). In addition, the number of MRSA detected in blood cultures (MRSAB, one isolate per patient case) and the number of *S. aureus* (both methicillin-susceptible and –resistant, one isolate per patient case) in blood cultures is collected.

Data analysis: The following parameters are analyzed: (i) screening rate (nasopharyngeal swabs for MRSA/100 inpatients), (ii) MRSA incidence (MRSA cases/100 inpatients), (iii) percentage of MRSA isolates per all *S. aureus* isolates detected in blood cultures, (iv) incidence density of MRSA isolates detected from blood cultures (MRSAB cases/100,000 patient days), (v) nosocomial MRSA incidence density (nosocomial MRSA-cases/1,000 patient days), (vi) length of stay in hospital (number of patient-days/inpatients , (vii) length of stay in the hospital of MRSA patients (number of patient-days of MRSA cases/ MRSA cases) are yearly calculated and reported to the participating hospitals. The validity of data included in the public health report is checked by the coordination center by plausibility checks (comparison to previous reports, comparison to data from quality reports of the respective hospitals), as well as the local public health authorities (comparison with mandatory reporting data for MRSA bacteremia cases). In case of implausible public health reports, the respective hospitals are contacted and the reports are checked for potential errors.

References:

(1) European Centre for Disease Prevention and Control, Antimicrobial resistance surveillance in Europe 2016. Annual Report of the European Antimicrobial Resistance Surveillance Network (EARS-Net). [cited 2019 Feb 03]. Available at: https://ecdc.europa.eu/sites/portal/files/documents/AMR-surveillance-Europe-2016.pdf.2017
